# Obstetric Outcomes of Nighttime Versus Daytime Delivery with Labor Epidural: An Observational Retrospective Study

**DOI:** 10.3390/jcm13175089

**Published:** 2024-08-27

**Authors:** Stefano Catarci, Bruno Antonio Zanfini, Emanuele Capone, Mariangela Di Muro, Luciano Frassanito, Giovanni Maria Maddaloni, Antonio Lanzone, Gaetano Draisci

**Affiliations:** 1 Department of Scienze Dell’ Emergenza, Anestesiologiche e Della Rianimazione, IRCCS Fondazione Policlinico A. Gemelli, 00168 Rome, Italy; brunoantonio.zanfini@policlinicogemelli.it (B.A.Z.); emanuele.capone@policlinicogemelli.it (E.C.); mariangela.dimuro@policlinicogemelli.it (M.D.M.); luciano.frassanito@policlinicogemelli.it (L.F.); giovannimaria.maddaloni@policlinicogemelli.it (G.M.M.); gaetano.draisci@policlinicogemelli.it (G.D.); 2 Department of Scienze della Salute della Donna, del Bambino e di Sanità Pubblica, IRCCS Fondazione Policlinico A. Gemelli, 00168 Rome, Italy

**Keywords:** epidural, daytime, nighttime, labor, urgent cesarean section

## Abstract

**Background**: Variability in obstetric outcomes in terms of the number and type of deliveries related to the day–night cycle has been described in previous studies. This 11-year retrospective analysis explores the effects of nighttime versus daytime delivery with labor epidural on obstetric outcomes. **Methods**: Data on deliveries performed between 1 October 2008 and 1 October 2019 were collected and differentiated into daytime, occurring from 8:00 a.m. to 7:59 p.m., and nighttime deliveries, occurring from 8:00 p.m. to 7:59 a.m. of the following day. The data collected included the patient history and maternal and neonatal outcomes. **Results**: A total of 29831 patients were included in the analysis. A positive and statistically significant correlation between the number of cesarean sections (Odds Ratio 1.35; 95% confidence interval = 1.26–1.44; *p* < 0.001) and the number of vaginal operative deliveries (Odds Ratio 1.21; 95% confidence interval = 1.01–1.44; *p* < 0.05) in patients who did not receive an epidural at nighttime was reported. Regarding the labor epidurals, a significantly greater incidence of accidental dural punctures with needles (0,4%; *p* < 0.05) in the nighttime versus daytime was reported. **Conclusions**: The absence of labor epidurals was associated with a significant increase in the number of cesarean sections and vaginal operative deliveries occurring at nighttime, without significant differences in labor duration. The incidence of anesthesiologic complications was greater in deliveries performed at nighttime.

## 1. Introduction

Variability in obstetric outcomes in terms of the number and type of deliveries related to the day–night cycle has been described in previous studies. Indeed, several studies have reported a variability in the number of non-elective deliveries related to the day–night cycle with a higher frequency of overnight births and a time-dependent variability in the efficacy of opioids and local anesthetics used for labor analgesia [1,2,3,4,5].

In a large retrospective analysis over a period of ten years, a significant difference in obstetric outcomes was reported, with night-onset labors having more vaginal deliveries, fewer cesarean sections, fewer labor augmentations with oxytocin, fewer artificial rupture of membranes, and a significantly shorter mean first stage duration. The authors found that labors starting at night appear to be more efficient than labors starting during the day [6].

Similarly, labor management differences and a variability with the time of day in the decision to perform cesarean sections were observed, particularly for dystocia, among nulliparous women who were attempting labor at term [7].

Furthermore, in previous studies, nighttime deliveries have also been associated with an increased risk of adverse perinatal outcomes [8,9].

The variability of non-elective births has been well described in the literature using a Poisson distribution. The Poisson distribution expresses the probabilities of a number of events that occur successively and independently in a given time interval, knowing that, on average, a number of them do occur. Specifically, the number of non-elective cesarean sections varies considerably over the days of the week and over the year. However, for each fixed day of the week, the variation is well described by the Poisson distribution, allowing for a simple prediction of the variability. Consequently, the Poisson model is suitable for estimating the variation in non-elective births and could be used for staff planning in obstetric clinics and to minimize healthcare costs [10].

Similarly, other studies have described variations in obstetric outcomes related to the day–night cycle by highlighting the potential effects of the circadian rhythm on labor. Chronological variations may be of critical importance for pregnancy and labor; one of the most important mechanisms affecting the labor process described in the literature is the release and timing of fetal–maternal hormones [11,12,13,14,15].

It should be recognized that the performance of anesthesiologists may also vary with the day–night cycle, potentially affecting clinical outcomes [16]. One of the primary differences between nighttime and daytime labor epidurals is the availability of healthcare providers: some hospitals during the day will typically have a full staff of anesthesiologists and nurses available, while at night, the availability of anesthesiologists may be more limited.

In the literature, data concerning the impact of labor epidurals on obstetric outcomes in deliveries occurring at nighttime compared with those in daytime are scarce.

The primary objective of this observational retrospective cohort study was to evaluate the impact of the day–night cycle on deliveries with labor epidurals on obstetric outcomes, assessed as the number of cesarean sections, number of vaginal operative deliveries (instrumental assistance with vacuum cup), and duration of labor.

The secondary objective of this study was to assess the incidence of epidural complications (accidental dural puncture, accidental vascular puncture) at night compared with those occurring in the daylight hours.

## 2. Materials and Methods

### 2.1. Study Population and Data Collection

This observational retrospective cohort study was carried out in the delivery room of the Fondazione Policlinico A. Gemelli IRCCS, Rome, Italy. This study received approval for data extraction and processing from an independent ethics committee without ad hoc consent from the enrolled patients (Policlinico A. Gemelli Ethics Committee, protocol ID 3741). This was a retrospective analysis of data collected from an electronic medical database that covered patient history and maternal and neonatal outcomes of all deliveries occurring between 1 October 2008 and 1 October 2019. Data regarding admission records, delivery medical records, partographs, and discharge codes from the International Classification of Diseases, Ninth Revision, Clinical Modification, were extracted.

The main maternal outcomes measured for the purpose of this retrospective analysis were as follows: number of non-elective cesarean sections (urgent cesarean sections), number of vaginal operative deliveries (instrumental assistance with vacuum cup), total duration of labor, first stage of labor duration, second stage of labor duration, and number of accidental vascular or dural punctures (with needle or catheter) when performing epidurals.

This article adheres to the applicable STROBE guidelines.

All deliveries during the period under review were examined and included in the analysis, except for elective cesarean sections, pregnancies with intrauterine fetal death occurring before the onset of labor and abortions (defined as the death of fetus prior to 22 weeks of gestation), births of infants with severe congenital malformations, and deliveries occurring outside the delivery room.

For this retrospective analysis, the delivery time was divided into two periods: daytime, from 8:00 a.m. to 7:59 p.m., and nighttime, from 8:00 p.m. to 7:59 a.m. on the following day. The choice of these cutoffs was made because of the rostering of medical personnel, as both doctors and residents followed this roster during the study interval.

In our tertiary center, the attending anesthesiologist is always present and dedicated to the delivery room (both at daytime and nighttime). Labor epidurals are performed by the attending anesthesiologist or by the resident in the presence of the anesthesiologist. The same organization is followed by obstetricians.

In accordance with institutional protocols, the suitability of performing labor epidural analgesia was assessed by an anesthesiologist 4–6 weeks before delivery based on the patient’s history, physical examination, and blood tests.

Patients with absolute (refusal of the patient, coagulopathy, increased intracranial pressure, sepsis or local infection at the site of puncture) or relative (aortic stenosis, anatomical deformities of the spine) contraindications were reassessed for epidural during labor and rejected, if appropriate; in patients receiving low-molecular-weight heparin, a withdrawal interval of ≥12 h was required if prophylactically administered, and an interval of ≥24 h was required if therapeutically administered, according to international guidelines. If analgesia was requested by the patient, after maternal non-invasive hemodynamic parameters and fetal well-being assessment, an infusion of crystalloids (5–7 mL/kg) was started and an epidural catheter was placed at the L2–L3 or L3–L4 intervertebral space. Analgesia was achieved by epidural administration of local anesthetics and fat-soluble opioids (0.1% ropivacaine 20 mL plus sufentanil 10 μg). Maternal blood pressure, heart rate, pain scores assessed by a visual analog scale (VAS), and the extent of sensory blockade were assessed at 5 min intervals for the first 15 min and approximately every hour thereafter; fetal well-being was monitored continuously via cardiotocography.

Analgesia was maintained with a manual “top-up” technique (administration of an additional dose of ropivacaine varying from 0.1% 20 mL to 0.15% 15 mL or 0.2% 10 mL, on the basis of relative pain, stage of labor, and extent of sensory blockade). When necessary, analgesia was supplemented with 6–10 mL of 2% lidocaine after secondment. This institutional protocol for achieving labor epidural was unchanged during the time interval of the study and was applied to all enrolled patients.

### 2.2. Statistical Analysis

Data in the manuscript were reported as the mean (±standard deviation) or count (percentage) as appropriate. Comparisons of the continuous variables night vs. day were tested with the Mann–Whitney test, having previously been tested for normality with the Shapiro–Wilk test. Associations were tested with the chi or Fisher exact test, when necessary. Frequency comparisons were performed with the z-test for proportions.

Statistical significance was considered with a *p* value of ≤0.05.

All analyses were performed with SAS v. 9.4 (SAS Institute, Cary, NC, USA).

## 3. Results

**Figure 1:**
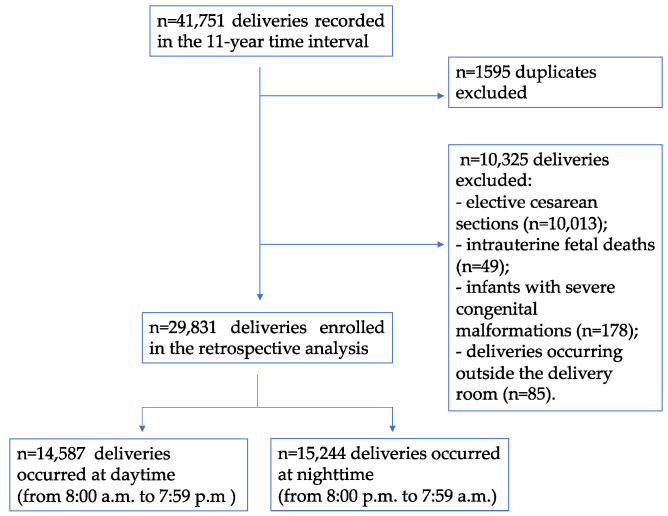
Flowchart of the study.

During the 11-year time interval, a total of 41,751 patients were screened for eligibility and included at baseline, as presented in Figure 1.

After the removal of 1595 duplicates, which were attributed to the repetition of data for each newborn of twin births, the information of 40,156 patients was reviewed. After the exclusion of 10,325 patients according to the exclusion protocol criteria, 29,831 patients were finally included in the analysis. The patients enrolled in the study were retrospectively divided into two groups: 14,587 deliveries (48.9%) occurring during the daytime, and 15,244 (51.1%) occurring at night. A total of 13,605 patients (45.6%) underwent a labor epidural.

The baseline characteristics of the study population and the obstetric and neonatal outcomes, retrospectively differentiated into day and night cohorts, are presented in Table 1.

No statistically significant differences were found in the total number of labor epidurals, vaginal operative deliveries (instrumental assistance with vacuum cup), or urgent cesarean sections performed at nighttime versus daytime.

The major indications for urgent cesarean sections were fetal distress, dystocia and fetal malposition.

Likewise, no significant differences in the length of hospital stay or neonatal Apgar scores were detected in the deliveries that occurred at night compared with those that occurred during the day.

Regarding the primary aim of our study, a positive and statistically significant correlation between the number of cesarean sections (OR, 1.35; 95% CI, 1.26–1.44; *p* < 0.001) and the number of vaginal operative deliveries (OR, 1.21; 95% CI, 1.01–1.44; *p* < 0.05) in patients who did not receive labor epidurals at nighttime versus daytime was reported.

On the other hand, no statistically significant differences in the number of cesarean sections or vaginal operative deliveries were detected between the patients who underwent labor epidurals at nighttime versus daytime.

Moreover, we reported a weak negative correlation for the total duration of labor in the night cohort compared with that in the day cohort, both in patients who underwent labor epidurals and in those who did not undergo epidurals (Table 2).

Referring to the incidence of anesthesiologic complications during labor epidurals, we identified a significantly greater incidence of accidental dural punctures with needles (0,4%; *p* < 0.05) in deliveries occurring at nighttime (Table 3).

## 4. Discussion

The administration of labor epidurals is a common practice that provides pain relief effectively and safely.

One intriguing aspect of this procedure is the timing of its management, specifically the difference between nighttime and daytime administration. The timing of epidural management and the timing of delivery itself may potentially influence maternal outcomes; this 11-year retrospective analysis explores the effects of nighttime versus daytime delivery with labor epidurals on obstetric outcomes.

The main finding of our analysis is that the absence of labor epidurals in nighttime delivery is associated with a statistically significant increase in the number of cesarean sections and vaginal operative deliveries, with the total duration of labor substantially unaffected.

The positive and statistically significant correlation between the lack of labor epidurals and the cesarean section rate is consistent with data from recent literature on the protective effect of epidural analgesia on the risk of cesarean sections [17,18,19,20].

Anim-Somuah M et al. analyzed 33 randomized controlled trials and concluded with moderate-quality evidence that labor epidurals had no impact on the risk of cesarean sections [17].

One hypothesis that could explain the apparently protective role of labor epidurals in decreasing the number of cesarean sections is the association between epidurals and the administration of oxytocin during labor (labor augmentation) to promote spontaneous vaginal delivery. Therefore, a negative association would reflect not only the direct effect of the epidural but also its relationship in clinical practice with labor augmentation, which usually results in spontaneous vaginal delivery [21,22].

Interestingly, no significant difference was found in the number of total urgent cesarean sections and vaginal operative deliveries (with or without labor epidurals) performed at nighttime versus daytime. Moreover, considering the number of anesthesiologic procedures performed on each shift as the anesthesiologist’s workload, the odds ratio of non-elective cesarean sections is not related to the anesthesia workload for the individual half-day period.

Likewise, the positive and statistically significant association between the absence of labor epidurals and vaginal operative deliveries in our patients at night appears to be noteworthy. Although Anim-Somuah M et al. reported an overall increase in assisted vaginal births with epidural analgesia [17], modern approaches to labor epidurals have been shown to abolish this effect [18].

Furthermore, our data suggest that labor epidural analgesia is not associated with a substantial increase in the total duration of labor in the two cohorts of patients studied. Wang et al. reported no significant difference in the duration of the first or second stage of labor or the rate of instrumental birth between the patients who did and did not undergo epidurals [23]. Instead, Cambic established that the use of epidurals is associated with a prolonged second stage of labor and that effective second-stage analgesia might also be associated with an increased rate of instrumental vaginal delivery [24].

Evaluating the role of individual healthcare providers in each shift, our findings are not attributable to a few individuals: a Bayesian analysis was conducted and showed that there was no significant variation in the number of non-elective cesarean sections due to the conduct of individual anesthesiologists. Additionally, the anesthesiologic protocols for performing labor epidurals were substantially unchanged during the 11-year interval of study.

Although this was not the aim of our study, no significant differences in neonatal well-being were detected among the deliveries occurring at nighttime or during the day, with or without epidurals, in our population of study.

Remarkably, the incidence of complications of neuraxial blocks in terms of accidental dural punctures was greater in the nighttime deliveries than in those performed during the daylight hours.

Several studies indicate that physical fatigue may negatively impact the performance of healthcare professionals and the outcomes of various medical procedures, resulting in an increased risk of complications [25,26]. A study of approximately 30,000 cases analyzed the number of deaths after elective surgery in relation to the day of the week, finding a higher mortality rate for patients undergoing operations on Fridays and weekends, with a 1.09-point increase in mortality each day after Monday and an 82% higher risk of death in the case of surgery performed on the weekend [27].

Additionally, the availability of more experienced anesthesiologists during the day may increase the precision of epidural placement, potentially reducing the risk of complications. A recent survey revealed how nighttime practice negatively affects anesthesiologist performance and may expose patients to anesthesia-related risks [16].

Notably, another potential benefit of daytime epidurals is improved communication and coordination among healthcare providers in various aspects of labor management. Increasing communication between team members has been emphasized as a means of reducing errors in obstetrics.

Childbirth is a complex clinical service requiring the coordinated support of highly trained healthcare professionals as well as the management of a finite set of critical resources (such as staff and beds) to provide safe care. Describing and analyzing the variability of deliveries over the day–night cycle may enable the construction of mathematical models to improve the organization of obstetrics units, reorganize staffing, and increase patient satisfaction. Some authors have proposed a queueing theory analysis to optimize resource management decisions and gauge appropriate anesthesia operating room staffing for maternity care. The queueing theory is a mathematical concept used for the study of congestion and delays arising from waiting in lines. It can help healthcare stakeholders create safe, efficient, and cost-effective workflow systems, such as in mass casualty events [28,29,30,31,32]. In the context of obstetrics, queueing theory analysis has been used to identify inefficiencies, optimize resource allocation among healthcare providers, reduce wait times for both scheduled and urgent cesarean deliveries or other time-sensitive activities, and ultimately improve the overall patient experience [28,29].

The retrospective and monocentric nature of this study can be considered its most important limitation. Studying the clinical practice of a single tertiary care center may have affected the results of this research. On the other hand, the large number of patients enrolled and the considerable time interval of the study allowed us to obtain highly significant findings.

Additional prospective observational studies will be needed to better describe the variability of obstetric outcomes in relation to the day–night cycle and labor epidurals. A better understanding of these events would allow for the optimization of the human resources available in delivery rooms and for an improved process of care provided in obstetric units.

## 5. Conclusions

This large retrospective analysis revealed that the absence of labor epidurals is associated with a statistically significant increase in the number of cesarean sections and vaginal operative deliveries occurring at night compared with those occurring in the daytime, without a substantial difference in the duration of labor.

The incidence of anesthesiologic complications in terms of accidental dural punctures with needles is greater in deliveries occurring at nighttime.

## Figures and Tables

**Table 1:** Baseline characteristics of the study population and obstetric and neonatal outcomes, retrospectively differentiated into day and night cohorts.

	Total Deliveries	Daytime	Nighttime	*p* Value
Age (years), n (SD)	33.1 ± 5.4	32.7 ± 5.4	32.5 ± 5.3	ns
Gestational age (weeks), n (SD)	39.3 ± 2.4	39.4 ± 2.5	39.5 ± 2.3	ns
Gravidity, n (SD)	2.0 ± 1.2	1.9 ± 1.2	1.9 ± 1.2	ns
Parity, n (SD)	0.4 ± 0.8	0.4 ± 0.8	0.5 ± 0.8	ns
Height (cm), n (SD)	164.3 ± 7.0	164.4 ± 6.7	164.3 ± 7.1	ns
Weight (kg), n (SD)	74.5 ± 15.2	74.1 ± 12.6	74.5 ± 12.7	ns
Weight increase (kg), n (SD)	12.5 ± 5.3	12.6 ± 5.3	12.7 ± 5.2	ns
Labor onset, Spontaneous, n (%)	20,285 (68)	9919 (68)	10,366 (68)	ns
Pregnancy complications, n (%)				
Gestational diabetes mellitus	2655 (8.9)	1283 (8.8)	1372 (9.0)	ns
Hypertensive disorders	985 (3.3)	490 (3.3)	495 (3.2)	ns
Labor epidurals, n (%)	12,976 (43.5)	6127 (42.0)	6849 (44.9)	ns
Urgent cesarean sections, n (%)	7300 (24.5)	4059 (27.8)	3241 (21.3)	ns
Operative vaginal deliveries, n (%)	1716 (5.7)	860 (5.9)	856 (5.6)	ns
Length of hospital stay (days), n (SD)	5.0 ± 3.9	4.7 ± 3.5	4.8 ± 2.8	ns
Neonatal weight (kg), n (SD)	3074.5 ± 661.7	3109.1 ± 663.2	3157.5 ± 609.3	ns
Neonatal Apgar 1’, n (SD)	8.5 ± 1.2	8.5 ± 1.2	8.6 ± 1.1	ns
Neonatal Apgar 5’, n (SD)	9.4 ± 1.0	9.5 ± 1.1	9.5 ± 1.0	ns

Data are expressed as mean ± standard deviation or count (percentage) as appropriate; ns = not significant (*p* value > 0.05).

**Table 2:** Obstetric outcomes in nighttime vs. daytime cohort with or without epidurals.

	Odds Ratio (95% IC) Labor Epidural		Odds Ratio (95% IC) Lack of Labor Epidural	*p* Value
Cesarean section	0.96 (0.86–1.06)	ns	1.35 (1.26–1.44)	<0.001
Vaginal operative delivery	1.09 (0.97–1.22)	ns	1.21 (1.01–1.44)	<0.05
Total duration of labor	0.99 (0.99–0.99)	<0.001	0.99 (0.99–0.99)	<0.001
Stage 1 labor duration	1.00 (1.00–1.00)	<0.05	1.00 (1.00–1.00)	<0.05
Stage 2 labor duration	0.99 (0.99–1.00)	<0.05	1.00 (1.00–1.00)	<0.05

ns = not significant (*p* value > 0.05).

**Table 3:** Frequency of anesthesiologic complications of epidurals in daytime and nighttime cohorts.

	Daytime	Nighttime	*p* Value
Accidental dural puncture, n (%)			
Needle	38 (0.3)	60 (0.4)	<0.05
Catheter	18 (0.1)	14 (0.1)	ns
Accidental vascular puncture, n (%)			
Needle	4 (0.03)	12 (0.1)	ns
Catheter	128 (0.9)	144 (1.0)	ns

ns = not significant (*p* value > 0.05).

## Data Availability

Data are unavailable due to privacy or ethical restrictions.
